# A comparative study of Barron's rubber band ligation with *Kshar Sutra* ligation in hemorrhoids

**DOI:** 10.4103/0974-7788.64407

**Published:** 2010

**Authors:** Rakhi Singh, Ramesh C. Arya, Satinder S. Minhas, Anil Dutt

**Affiliations:** *Medical Wing, Savera Association, Sreeniwaspuri, New Delhi - 110 065, India*; 1 *Department of Shalya, RGGPGAC, Paprola (H.P.), India*; 2 *Department of General Surgery, I.G.M.C., Shimla (H.P.), India*

**Keywords:** Barron's rubber band ligation, hemorrhoids, *kshar Sutra* ligation

## Abstract

Despite a long medical history of identification and treatment, hemorrhoids still pose a challenge to the medical fraternity in terms of finding satisfactory cure of the disease. In this study, *Kshar Sutra* Ligation (KSL), a modality of treatment described in Ayurveda, was compared with Barron's Rubber Band Ligation (RBL) for grade II and grade III hemorrhoids. This study was conducted in 20 adult patients of either sex with grade II and grade III hemorrhoids at two different hospitals. Patients were randomly allotted to two groups of 10 patients each. Group I patients underwent RBL, whereas patients of group II underwent KSL. *Guggul*-based *Apamarga Kshar Sutra* was prepared according to the principles laid down in ancient Ayurvedic texts and methodology standardized by IIIM, Jammu and CDRI, Lucknow. Comparative assessment of RBL and KSL was done according to 16 criteria. Although the two procedures were compared on 15 criteria, treatment outcome of grade II and grade III hemorrhoids was decided chiefly on the basis of patient satisfaction index (subjective criterion) and ability of each procedure to deal with prolapse of internal hemorrhoidal masses (objective criterion): Findings in each case were recorded over a follow-up of four weeks (postoperative days 1, 3, 7, 15 and 30). Statistical analysis was done using Student's *t* test for parametric data and Chi square test & Mann-Whitney test for non-parametric data. *P* < 0.05 was considered significant. RBL had the advantages of being an OPD procedure requiring no anesthesia and was attended by significantly lesser postoperative recumbency (*P* < 0.001 ) and significantly lesser pain (*P* < 0.005 on day 1) as compared to KSL. However, Group II (KSL) scored better in terms of treatment outcome. In Group II, there was significantly high (*P* < 0.05) patient satisfaction index as compared to Group I. Group II reported 100% 'cure' (absence of hemorrhoidal masses even on proctoscopy) of internal hemorrhoidal prolapse as against 80% in Group I (RBL); however, this difference was statistically insignificant (*P* > 0.05). Both the groups were comparable statistically on all other grounds. *Kshar Sutra* Ligation is a useful form of treatment for Grades II and III internal hemorrhoids.

## INTRODUCTION

Hemorrhoids have plagued human civilization for centuries. Owing to the high prevalence of the disease, researchers have come up with a wide spectrum of treatment modalities, operative as well as non-operative. Rubber Band Ligation (RBL) is a well-known technique of internal hemorrhoidal ligation. *Kshar Sutra* Ligation (KSL) is an Ayurvedictechnique of internal hemorrhoidal ligation with medicated thread called *Kshar Sutra*. *Kshar Sutra* literally means a thread smeared with *Kshar*; *Kshar* in Sanskrit means 'one capable of violence / destruction'.[1] *Kshar Sutra* Ligature performs excision by virtue of its mechanical pressure and phytochemical cauterization. This study aims at comparative assessment of RBL with KSL of internal hemorrhoids of second or third degree.[2]

## MATERIALS AND METHODS

After obtaining permission from Ethics Committee, twenty adult patients of either sex with symptomatic grade II or grade III hemorrhoids were selected from the OPDs. Patients excluded from the study were those unfit for anesthesia; patients with grade I and grade IV hemorrhoids; concomitant anorectal conditions like anal fissure, fistula in ano, colitis, malignancy; pregnant patients; those with proven immunodeficiency; patients with diabetes mellitus, tuberculosis and obstructive uropathies.

Twenty patients were divided into two groups of 10 patients each. Group I were treated with RBL while group II received KSL using *Guggul*-based *Apamarga Kshar Sutra*. The procedures were done in two different hospitals.

Written informed consent was taken from all the patients prior to embarking on the examination and treatment.

Findings in each case were recorded over a follow-up of four weeks on postoperative days 1, 3, 7, 15 and 30.

In the present study, *Guggul*-based *Apamarga Kshar Sutra* were prepared using the methodology based on the description of *Kshar Sutra* in the Ayurvedic texts of *Sushruta Samhita*,[1] *Rasakamadhenu*,[3] *Bhaishajya Ratnavali*[4] and *Rasatarangani*,[5] and in keeping with the specifications laid down by IIIM, Jammu and CDRI, Lucknow.[6]

### Preparation of Guggul-based Apamarga *Kshar Sutra*

**Figure 1:**
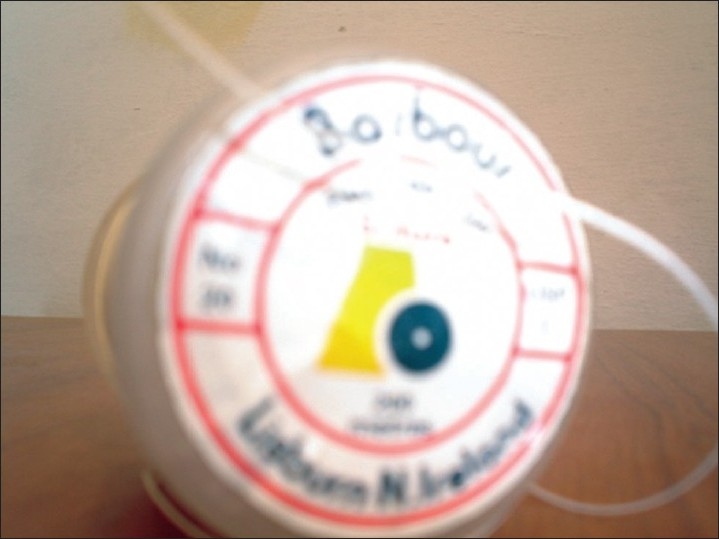
Barbour's linen

- **Requirements**: [Figure 1]
  1. **Thread**: Surgical linen thread of size 20 (having a tensile strength of 5.0 kg) was chosen.
  2. ***Guggul (Commiphora mukul)***: *Guggul* (resin) was procured and kept in absolute alcohol (ethanol) so that a sticky solution of *Guggul* in was formed.
  3. **Haridra (*Curcuma longa*)**:Turmeric or *haridra* was obtained by grinding dried rhizomes of *Curcuma longa* to a fine powder (collected by passing through sieve number 100).
  4. ***Apamarga Kshar***: This *Kshar* was prepared from *Achyranthes aspera* collected during September / October, 04 when the plant was in fruiting stage. The whole plants were allowed to dry in shade. The pile of dry plants was put to fire and ashes were allowed to cool down. Ashes were collected in a stainless steel vessel and stirred thoroughly in water (1 : 5 w / v) and set aside. The floating mass of wood charcoal. etc., was skimmed off the surface, the supernatant decanted and filtered. The process of extracting the ash with water was repeated five times and the combined filtrate evaporated in stainless steel vessel. The dried residue, which was meticulously stirred so as not to get over cooked or burnt, constituted the desired *Apamarga Kshar*. This was ground to a fine powder (#100) and packed in air tight sealed containers. The yield of *Apamarga Kshar* from the total ash of the plant was between 40–42% w / w.

**Figure 2:**
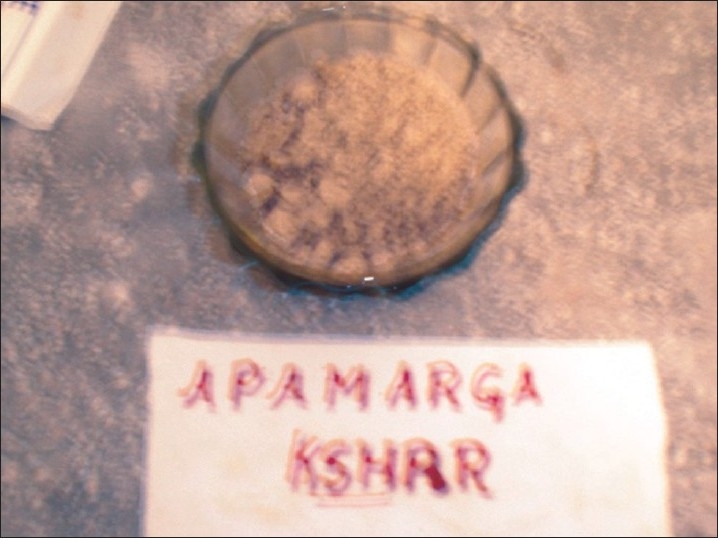
Apamarga Kshar

***Kshar Sutra* Cabinet**: Vertical design *Kshar Sutra* Cabinet was constructed .from hardboard and the inside of the cabinet was lined by unpainted aluminum sheets all over so that the light and heat could be reflected from metallic surfaces. The front of the *Kshar Sutra* Cabinet was made of glass to facilitate inside view [Figure 2].

**Figure 3:**
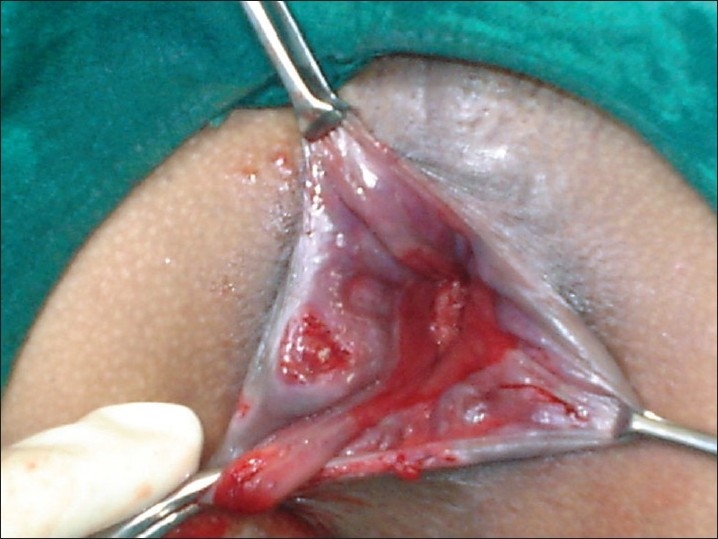
Triangle of exposure with internal hemorrhoidal mass

The *Kshar Sutra* hangers could be slid inside like trays, on the supports constructed for the purpose. The Cabinet was provided with an ultraviolet tube for the purpose of sterilization of *Kshar Sutra*. The control of these electrical appliances, namely U-V tube and two bulbs was fixed outside the *Kshar Sutra* Cabinet [Figure 3].

Hot air blowers to dry the coatings on *Kshar Sutra* and silica bags as dessicants could be installed and used as and when required.

### Technique of preparation

Surgical linen thread no. 20 was tied to one end of the hanger and hooked to all the notches till the other end of the hanger was reached where it was tied snugly once again.

The threads were then smeared uniformly on all sides (front, back, above and below) with *Guggul* solution (in absolute ethanol) with the help of a clean gauze soaked in *Guggul* solution. The hanger was then replaced in the Cabinet, which was was closed properly and threads dried with the help of two 200 W bulbs and / or hot air blower. The same process was repeated 11 times. After each coating U-V light exposure was given to the threads for 20–30 min daily.

The 12^th^ coating was done by first smearing the thread with *GuggulGuggul* solution and then passing the wet thread through a heap of finely powdered *Kshar*. When all the threads were smeared with *Kshar*, the hanger was gently shaken so that the excess *Kshar* particles fell down. The hanger was now replaced in the Cabinet for drying and for U-V light exposure. This process was repeated till seven coatings of *Guggul* and *Kshar* were achieved.

**Figure 4:**
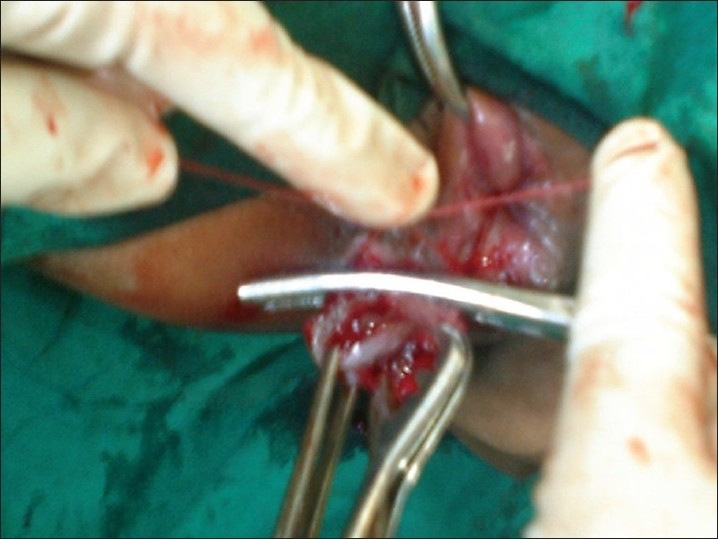
Transfixation and ligation of hemorrhoids with *Kshar Sutra*

The remaining three coatings were finally completed with *Guggul* solution and fine powder of turmeric. *Guggul* solution was applied gently and uniformly over the threads and then the wet threads were passed through a heap of fine turmeric (*haridra*) powder. After each coating the threads were dried in the Cabinet and given U-V light exposure for 30 min [Figure 4].

Thus, 21 coatings over the threads were completed. The order of coatings can be summarized as follows:

*Guggul* solution 11

*Guggul* solution + *Apamarga Kshar* (alternately) 7 each

*Guggul* solution + *Haridra powder* (alternately) = 3 each

Total coatings = 21

**Figure 5:**
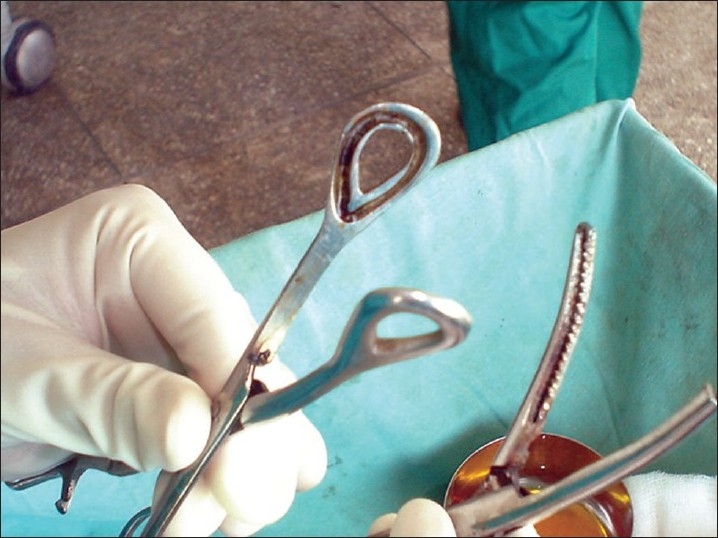
Self-improvised pile mass crushing forceps

During the manufacturing process, sterilization was maintained by using 'no touch technique' and giving appropriate ultraviolet light exposure.[7] One of the prepared *Kshar Sutras* was cultured to rule out any microbial growth and it was found to have no fungal and minimal bacterial growth [Figure 5].

### Technique of *Kshar Sutra* ligation in hemorrhoids

#### Preoperative preparation

Bowel preparation was done with soap water enema and patients were kept fasting for 12 h before the surgery.

#### Operative steps

**Figure 6:**
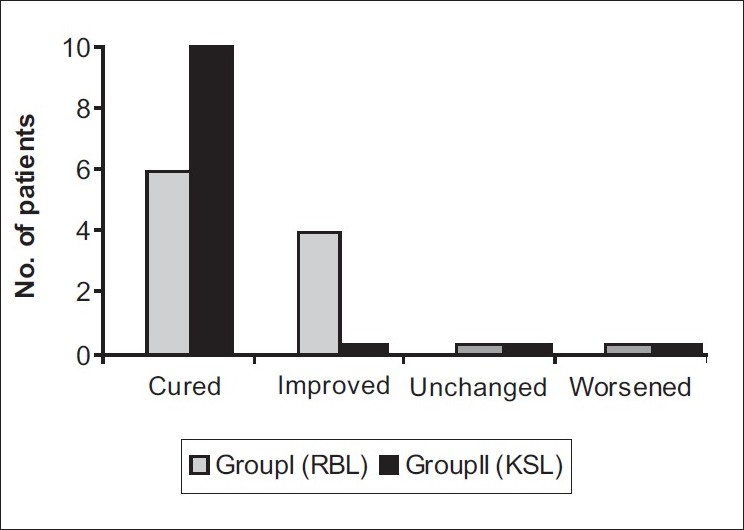
Patient Satisfaction

**Figure 7:**
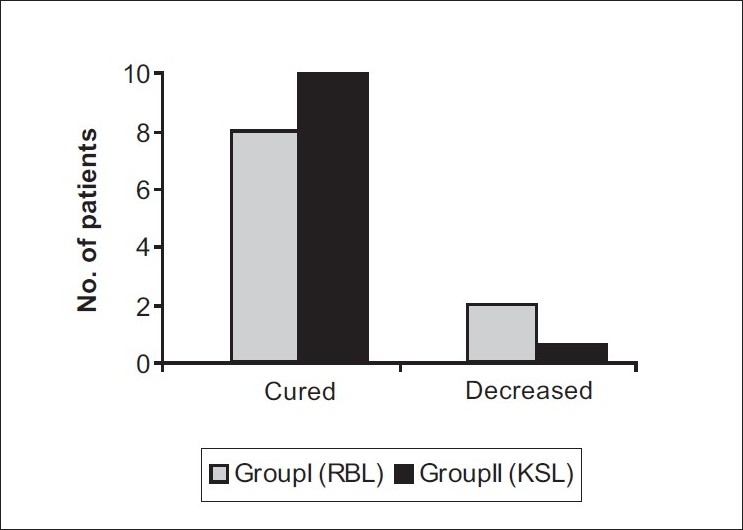
Prolapse of internal haemorrhoids treatment outcome

1. Sedation was given with Diazepam 10 mg, and Pentazocine 30 mg intravenouslyslowly. The patient was placed in a Lithotomy position. A hemorrhoidal plexus block and / or pudendal block with liberal local infiltration of 1% lignocaine was given thereafter. The hemorrhoidal masses and their positions were identified by a thorough proctoscopic examination. Gentle digital anal stretching of no more than six (3+3) fingers (Recamier / Maisonneuve procedure)[8,9] was done. Intracanal packing was given to avoid soiling during operation.
2. Revelation of the 'Triangle of Exposure'[2]: The skin-covered component of each of the main piles was seized with Allis tissue holding forceps usually in the 3, 7 and 11 o'clock positions and retracted outwards, keeping the Allis tissue forceps in anatomical positions. This revealed the 'Triangle of Exposure' i.e. the mucosal component or internal hemorrhoids at the upper pole and dermal component in the grip of Allis forceps at the lower pole. The left lateral internal pile was grasped with a pile mass holding forceps. The two forceps attached to left lateral pile were taken in the palm of left hand drawn outwards and left index finger was used to stabilize the inner aspect of the pile mass [Figure 6].
3. A semi-circular groove was made with blunt scissors in the anal and perianal skin including the external part of hemorrhoids held with Allis tissue forceps. The incision reached the mucocutaneous junction but did not extend into the mucosa. All this while was done with the index finger firmly pressed against the scissors and traction maintained so that lower edge of internal sphincter was preserved and not included in the tissue of transfixation. Without carrying out further dissection, the pile mass crushing forceps was applied to the external and internal pile components were held with two forceps. The pile masses were twisted and the pedicle of pile mass was crushed within the jaws of the pile mass crushing forceps until it assumed a thinned-out shape.
4. The pile mass was transfixed and ligated by passing a 76 mm round body curved needle loaded with the *Kshar Sutra* through the base of the crushed pedicle, and transfixing and ligating both the external and internal parts of the hemorrhoidal mass with *Kshar Sutra* [Figure 7]. The hemorrhoidal mass was excised about 5 mm distal to ligature and *Kshar Sutra* left *in situ* for further inspection. The right posterior and last of all, right anterior hemorrhoidal masses were dealt within the same manner. Hemostasis ensured, the transfixation *Kshar Sutra* was divided to a short length. Gauze soaked in lignocaine jelly was placed inside the canal. One end of anal packing given initially was withdrawn out for facilitating easy removal and a T-bandage was given.

### Postoperative management

Paracetamol was given to patients on demand for analgesia and patients were allowed oral intake 3 h after surgery. The dressing was removed the next morning after a lukewarm sitz bath. =Bulk evacuant (Isabgol) was prescribed and a light diet was advised till first bowel movement.[10]

Sloughing off of hemorrhoidal masses with automatic removal of *Kshar Sutras* and wound healing were monitored daily till discharge of patient from hospital.

### Technique of rubber band ligation

#### Preoperative preparation

A glycerine enema was administered preoperatively.

### Operative steps

The patient was placed left lateral position. The inside of the proctoscope was illuminated by inbuilt or external light source.

#### Technique of application[11]

The proctoscope was passed through the anal canal into the rectum and the hemorrhoidal masses were identified. While an assistant held the handle of proctoscope, the Barron's ligator was introduced through the proctoscope and brought to bear on the mucosal part of the most prominent pile. The long alligator forceps were next passed through the proctoscope and through the drum at the end of the ligator. The pile was seized with the forceps and drawn well into the drum, while the distal end of the drum was firmly pressed against the anal canal wall, special care was taken to see that its inferior edge was at least 1 cm above the pectinate line. If at this point, the patient complained of pain or discomfort, the mucosa was set free and grasped again a little higher up, away from the pectinate line. By closing the handles of the ligator, the rubber ring was pushed off the drum and instantly closed on the base of the pile; the resulting nub of strangulated tissue was about 1 cm in diameter. Multiple ligations were done in all the patients of group I.

### Postoperative follow-up

Patients were advised not to strain to avoid slippage of rubber band and oral paracetamol was given on demand by patient. Bulk evacuant and light diet was prescribed till sloughing off of the hemorrhoidal masses.

RBL and KSL were compared statistically using Student's 't' test on the following variables

Demographic data, predisposing factors, clinical presentation to ensure that the two groups were statistically comparable and to remove any bias. End points of treatment were defined as subjective and objective.

1. **Subjective**: Since hemorrhoids create a subjective problem in patients, cure of disease does not amount to merely restoration of anal canal anatomy. Patient satisfaction resulting from alleviation of symptoms and improved quality of life is equally desirable. Therefore, patient satisfaction index was taken as foremost point to define endpoint of treatment. All the patients were asked to express their degree of satisfaction about treatment in terms of 'cured' / 'improved' / 'unchanged' / 'worsened' at four weeks of follow-up after procedures.
2. **Objective**: The cardinal feature of hemorrhoids grade II and III is internal hemorrhoidal prolapse, which is spontaneously reducible in grade II and digitally reducible in grade III, respectively. Other features like bleeding, pain, may or may not be present. Complete cure refers to absence of internal hemorrhoidal prolapse even on proctoscopy, and failure of treatment means internal hemorrhoidal prolapse either remains unchanged or progresses from Golighers' grade II to III and from grade III to IV.

**Results of RBL and KSL were assessed according to 15 criteria as follows:**

1. Patient satisfaction (cured / improved / unchanged / worsened as expressed by patient at the end of follow-up of four weeks)
2. Prolapse of internal hemorrhoids: treatment outcome (cured / decreased / unchanged / increased).
  A prolapse of 0.5 to 2 cm of anal mucosa containing engorged hemorrhoidal plexus vessels was considered as prolapsed internal hemorrhoidal mass.
  After treatment, outcome was noted using following parameters:
  **Cured:** Absence of hemorrhoidal mass even on proctoscopy
  **Decreased:** Conversion of grade III (digitally reducible) hemorrhoids into grade II (spontaneously reducible): conversion of grade II (spontaneously reducible) hemorrhoids into grade I (visible only on proctoscopy).
  **Unchanged:** No change in size / reducibility of hemorrhoids.
  **Increased:** Conversion of grade II into grade III and grade III into grade IV (irreducible) hemorrhoids.
3. Hospital stay (in days).
4. Postoperative pain (recorded on Visual Analog Scale on days 1, 3, 7, 15 and 30 after operation).
5. Postoperative hemorrhage (recorded in milliliters on days 1, 3, 7, 15, 30 postoperatively).
6. Postoperative urinary complaints: If encountered, type of complaint (dysuria / retention urine) and treatment given along with day of occurrence.
7. Return to day to day activities ((in days).
8. Time taken for sloughing off of hemorrhoidal masses (in days)
9. Involvement of external hemorrhoidal plexus: treatment outcome (Not applicable / cured / decreased / unchanged / increased).
10. Postoperative discharge per anum: If encountered, nature of discharge (mucoid / purulent / mucopurulent), amount (ml approx.) and day of occurrence.
11. Wound infection (absent / present)
12. Subsequent procedure (RBL / Sclerotherapy / refashioning of external wound) and number of subsequent procedures required.
13. Anal incontinence (absent / present). If present, whether permanent or temporary and whether incontinence was of feces / flatus / both.
14. Anal stenosis (absent / present)
15. Recurrence at 04 weeks (present / absent)

## RESULTS

### Comparative assessment of RBL and KSL

**Figure 8:**
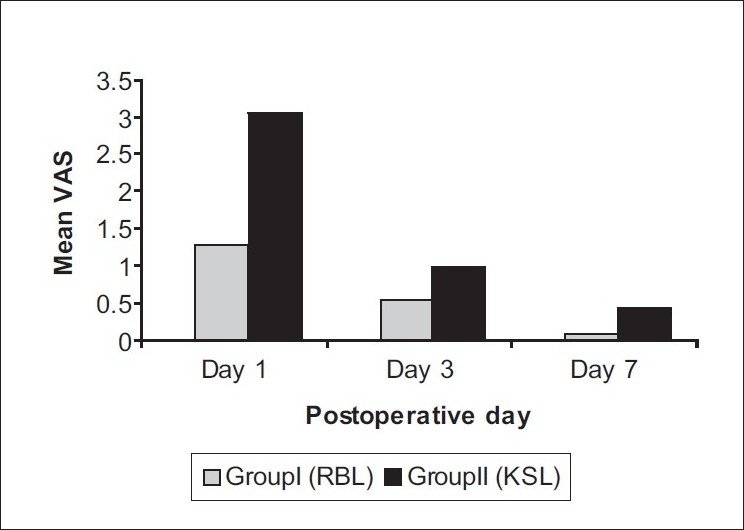
Postoperative Pain

**Figure 9:**
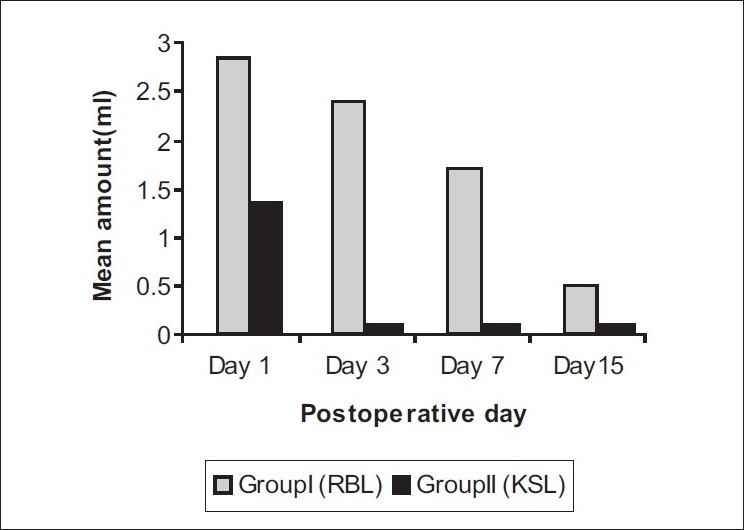
Postoperative Haemorrhage

1. **Patient satisfaction:** At 4 weeks of follow-up, all patients were asked to express their degree of satisfaction about treatment outcome in terms of 'cured' / 'improved' / 'unchanged' / 'worsened'. In group I (RBL), 40% patients reported as 'improved' and 60% as 'cured' while all patients in group II (KSL) reported as 'cured'. This difference of patient satisfaction was significant statistically at *P* < 0.05 using Chi-square test, with group II patients reporting a higher degree of satisfaction at 04 weeks [Figure 6].
2. **Prolapse of internal hemorrhoids (treatment outcome):** In group I (RBL), treatment outcome of internal hemorrhoids ranged from 'cure' (i.e. absence of hemorrhoidal masses even on proctoscopy) in 8) of the 10 patients to 'decrease' in prolapse (i.e. conversion of grade III into grade II hemorrhoids) in the remaining 2 patients. In group II (KSL), all 10patients reported 'cure' of internal hemorrhoidal prolapse. This difference was statistically insignificant with *P* > 0.05 using the Chi-square test [Figure 7].
3. **Hospital stay:** Group I (RBL) patients did not require hospital stay for more than an hour, whereas group II (KSL) patients had to stay in the hospital for mean period of 9.8 ± 4.6 days. This difference was statistically highly significant at *P* < 0.001 with group II patients requiring a comparatively longer hospital stay.
4. **Postoperative pain:** The pain scores of two groups as measured on VAS turned out to be significantly higher in group II than Group I on Day1, 3 and 7 (*P* < 0.005). On Day 30, no patient of either group experienced pain [Figure 8].
5. **Postoperative hemorrhage:** Proctorrhagia, measured in milliliters was comparable in the two groups on Days 1, 3, 7, 15 and 3. [Figure 9].
6. **Postoperative urinary complaints:** Dysuria and retention urine developed in 1patient each in group II (KSL) on Day 1, and dysuria was present in 1patient of group II on Day 3. No urinary complications were encountered in group I.
7. **Return to day to day activities:** In group I, majority of patients, i.e. 80% returned to their work the same day. Average time spent off work was 0.3 ± 0.55 days. In group II, average time spent off work was 3 ± 1.73 days. Statistically, group II patients had to spend significantly longer time in bed as compared to group I with *P* < 0.001.
8. **Time taken for sloughing off of hemorrhoidal masses:** In group I, average time taken for sloughing off of hemorrhoidal masses was 4.8 ± 1.4 days. In group II, mean time taken was 7.1 ± 0.62 days. The difference was statistically highly significant at *P* < 0.002 with group II patients taking longer for sloughing off of hemorrhoidal masses.
9. **Engorgement of external hemorrhoidal plexus (treatment outcome):** In group I, external hemorrhoidal plexus involvement remained 'unchanged' even after RBL. In group II, it was 'cured' completely after KSL.[Figure 5].
10. **Postoperative discharge:** Amount and nature of postoperative discharge on Days 1, 3, 7, 15, 30 in the two groups were comparable with insignificant difference at *P* > 0.05 .
11. **Wound infection:** One patient in group II developed wound infection.
12. **Subsequent procedure:** In group I, 4 patients and in group II 1 patient required subsequent procedures.

Shortly after RBL, overstraining by patient to overcome anal discomfort or foreign body sensation and application of elastic band in insignificant Grade-II hemorrhoids strangulating very small hemorrhoidal tissue resulted in slippage of Rubber Bands necessitating reapplication. In3 cases of slippage of Rubber Bands detected on Day 1 or Day 3, reapplication of elastic bands was done immediately. In 1 cases sclerotherapy was performed to check proctorrhagia, which was mild (<5 ml) yet problematic to the patient at 7 days of follow-up.

In group II, 1 patient had to undergo refashioning of external wound three weeks after *Kshar Sutra Karma*. This was done to remove remnant skin tag and facilitate anal hygiene.

Anal stenosis, anal incontinence, or recurrence were not encountered in any patient of either group during a follow-up of four weeks.

## DISCUSSION

This study was a pilot study conducted to compare rubber band ligation with *kshara sutra karma* to treat patients of hemorrhoids, using various subjective and objective methods for comparison.

Rubber Band Ligation relies on the principle of 'Mucosal Fixation'. The mucosa and the submucosal vascular cushions are fixed to the underlying muscle coat by creating scarring after full thickness ulceration. *Kashara sutra* transfixation and ligation leads to strangulation of the hemorrhoidal tissue.

By virtue of its penetrating and '*Ksharan*' properties, the *kshara* induces a sterile inflammatory response in hemorrhoidal tissue and neighboring tissue leading to scarring and mucosal fixation. *Guggul*, by virtue of its soothing action, prevents harm to delicate anal canal tissue by the irritant action of *Kshar*. *Haridra* by virtue of its antiseptic, *varnya* (wound healing) and *krimighna* (antimicrobial) properties promotes wound healing as well.

Since hemorrhoids is, primarily, a subjective disease presenting with a distressing symptom complex of pain, bleeding, prolapsed piles, discharge per anum, it is important that treatment is targeted at elimination of symptoms, improvement of quality of life and hence patient satisfaction. KSL scored higher on this parameter as compared to RBL. On the objective parameter of prolapse of internal hemorrhoids (treatment outcome) Group II gave better treatment outcome than group I. Since the difference in treatment outcome was insignificant statistically, we concluded that the two forms of treatment were comparable on this aspect [Figure 3].

In group I, all the patients underwent RBL without any anesthetic requirement. This was possible because RBL is done about 1 cm above dentate line in the base of hemorrhoids, strangulating rectal mucosa or upper anal canal mucosa, which is pain insensitive due to autonomic innervation. In group II, all the patients required local hemorrhoidal and/or pudendal block along with sedation. This facilitated Recamier's digital anal dilatation up to maximum of six fingers. Also pain sensitive external part of hemorrhoids could be dealt with as effectively as internal hemorrhoids.

In group II, statistically significantly higher pain score was observed as incorporation of external hemorrhoidal tissue within *Kshar Sutra* ligature caused pain after effect of local anesthesia and sedation had worn off. This pain responded well to simple analgesics like paracetamol, warm sitz bath, and ensuring that first bowel movement after *ksharasutra* ligation was soft and smooth. On the other hand, RBL produced less pain as RBL was done 1 cm above dentate line in pain insensitive mucosa, banding only the internal hemorrhoids.

The observed gradual control of bleeding in RBL as compared to KSL can be accounted for by the different principles of both these treatments. RBL reduces bleeding by producing mucosal fixation and submucosal fibrosis.[12] This process of submucosal fibrosis is completed by three weeks. Therefore, proctorrhagia is controlled by the end of three weeks. KSL reduces or cures proctorrhagia by ligation and phytochemical cauterization of hemorrhoidal mass or vascular cushions at the time of operation and so cure of proctorrhagia is evident soon after KSL.

KSL is an operative technique attended to by greater postoperative pain. Higher postoperative pain combined with factors like perfusion volumes over 1000 ml, use of pentazocine, multi-pedicular hemorrhoidectomy and neurotonic susceptibility of the patient resulted in postoperative urinary complaints. However, this complication was completely prevented after first two cases by taking precautions such as encouraging immediate preoperative urination, restricting perfusion volume to not more that 500 ml, avoiding large intracanal dressings or packings, hot fomentation and warm sitz bath (between 40° and 50°C), which have been shown to induce lowering of urethral pressure and micturition,[12] avoiding opioid analgesics in postoperative phase, and excluding patients with obstructive uropathies for KSL treatment RBL resulted in reduction of internal hemorrhoidal prolapse but not its complete cure in 2 patients. External hemorrhoidal plexus engorgement remained unchanged after RBL. Persistent internal hemorrhoidal prolapse and external hemorrhoidal plexus engorgement interfering with anal hygiene were the causes of per anal discharge. Helminthic infestation was also suspected for which deworming was done.

The average period of anal stenosis is six weeks, although it can be detected at four weeks. No incidence of anal stenosis was observed in either group in the present study. In Group II being the following precautions were taken to prevent anal stenosis, minimizing tissue dissection (in the form of semi-circular groove) thus safely preventing circumferential loss of anal canal mucosa, maintenance of normal intestinal transit by use of bulk evacuant postoperatively, regular monitoring of wound healing in order to avoid any wound stickiness, and single digital dilatation P/A starting from seventh postoperative day, until complete scarring.

In KSL, external hemorrhoidal plexus or dermal component was included in the ligature besides internal part of hemorrhoids. On the contrary, in RBL, only a part of internal hemorrhoidal tissue was included in elastic band ligature, the strangulated tissue mass being about 1 cm. Small tissue mass held in elastic ligature resulted in formation of a smaller wound by rapid sloughing off in group I as compared to group II.

Since RBL causes necrosis of only a small nub of internal pile, it was found to be ineffective in relieving or curing external hemorrhoidal plexus engorgement. KSL dealt with external hemorrhoidal plexus engorgement at the time of operation by including it in the transfixation ligature and thus resulting in its complete cure in all the patients.

In the present study, anal incontinence of any type (feces, flatus, both) was not encountered in any patient of group I and group II. Anal incontinence after KSL was prevented by taking following precautions: digital dilatation of only six (3+3) fingers was done by applying gentle, steady and continuous pressure in all directions and making circular finger movements for about 10 min,[8,9] internal anal sphincter was carefully prevented from being injured or included in *Kshar Sutra* ligature, sphincterotomy was not performed in any case,[13] wide retractors like Park's retractor or *Ferguson's* retractor were not used during surgery[14] and patients with history of obstetric anal trauma and neuropathic irritable bowel syndrome were excluded from the study.

This study had some limitations including the fact that this is not randomized study as two groups were treated in two different hospitals. Ten consecutive patients fulfilling inclusion criteria were included in each group. The sample size was small, but homogeneity was maintained in terms of demographic features, clinical features of patients and the team performing procedures. Follow-up of patients could have been longer than 04 weeks, but for the limitations imposed by time period within which this study was to be completed. However, this is the first study of its kind comparing KSL for hemorrhoids with a conventional technique like RBL, albeit in a small sample of patients. *Guggul* has sticking property. It also has *Vranashodhana, Vranaropana, Putihara, Jantughna* and *Vedanasthapana* properties. Due to the above facts, *Guggul* was used in the present study in place of Snuhi latex for making *Kshar Sutra*. Use of *Guggul*-based Apamarga *Kshar Sutra* in treating hemorrhoids grades II and III is new.

We have also modified the technique of KSL such as gentle anal stretching and three fingers Recamier's anal dilatation instead of the more traumatic Lord's procedure to prevent injury to internal anal sphincter, minimal tissue dissection in the form of semi-circular groove on anal skin, crushing and thinning of pile mass pedicle using specially improvised pile mass crushing forceps leading to less tissue trauma and negligible blood loss during the procedure, minimal tissue dissection saving circumferential loss of anal mucosa, andtight, secure ligature as *Kshar Sutra* is made from linen thread. We did not rely on *Kshar Sutra* procured from market. *Apamarga Kshar*, fully equipped *Kshar Sutra* Cabinet and *Guggul*-based *Apamarga Kshar Sutra* were manufactured in the department itself using standard techniques. This has helped us achieve better quality of cost effective *Kshar Sutras*.

One *Kshar Sutra* from the batch was subjected to culture and sensitivity test to rule out contamination. Physicochemical analysis of one *Kshar Sutra* was also performed to see that pH of *Kshar Sutra* was within standard range (pH was 10.6).

## CONCLUSION

Rubber Band Ligation (RBL) has the distinct advantage of being an OPD procedure, thus obviating the need for hospitalization and requires no anesthesia. Postoperative pain is significantly lesser. Time spent off work by the patient is also significantly lesser as compared to *Kshar Sutra* Ligation. However, RBL was not able to reduce or cure external hemorrhoidal engorgement, which was not only unaesthetic but also created problems like pruritus ani, mucoid discharge, peri-anal dermatitis and soiling of underclothes. RBL also showed less success in dealing with internal hemorrhoidal prolapse in grade III hemorrhoids.

KSL fared better as compared to RBL in terms of complete cure of symptoms like prolapse, mucoid discharge, external hemorrhoidal engorgement. This resulted in significantly greater satisfaction index in group II patients at the end of the treatment.
